# Acknowledgement to Reviewers of *Insects* in 2014

**DOI:** 10.3390/insects6010100

**Published:** 2015-01-08

**Authors:** Insects Editorial Office

**Affiliations:** MDPI AG, Klybeckstrasse 64, CH-4057 Basel, Switzerland

The editors of *Insects* would like to express their sincere gratitude to the following reviewers for assessing manuscripts in 2014:

Acebes, A.L.
Aguilar-Fenollosa, Tina
Anderbran, Olle
Anfora, Gianfranco
Anton, Sylvia
Ayasse, Manfred
Baker, Tomas
Belcari, Antonio
Bengtsson, Jonas M.
Biergans, Stephanie D.
Biondi, Antonio
Bloch, Guy
Bos, Nick
Breed, Michael
Cameron, Guy N.
Canale, A.
Chang, Cynthia C.
Chapman, Nadine
Cheng, Shing Kwong
Clark, Kenneth L.
Conti, Barbara
Cruse, Holk
Cunningham, Paul
Daniels, Jaret C.
Davies, Thomas G. Emyr
de Biseau, Jean-Christophe
de Ibarra, Natalie Hempel
Dembilio, Óscar
Devaud, Jean-Marc
Dionisio, Giuseppe
Doggett, Stephen
Dong, Ke
Duan, Jianjun
Duso, Carlo
Elkinton, JS
Espino, Luis
Evenden, Maja L.
Farris, Sarah
Feldlaufer, Mark
Ferveur, Jean-Francois
Fiene, Justin
Forister, Matthew
Foster, Stephen P.
Ganloff-Kaufmann, Jody
Gautam, Bal
Gibert, Patricia
Goddard, Jerome
Greenberg, Les
Groot, Astrid
Gross, Jürgen
Gruter, Christoph
Gubler, Duane
Guedot, Christelle
Gwiazdowski, Rodger
Harari, Ally
Harris, Marvin
Hassan, Errol
Hautier, Louis
Hébert, C.
Helmerhorst, Eva
Henderson, Gregg
Hodson, A.k.
Homberg, Uwe
Hoy, Marjorie
Hribar, Larry
Huang, Juan
Huang, Rong-nan
Jacups, Susan
Jing, Xiangfeng
Judge, Kevin
Kelber, Almut
Kim, Dong Soon
Knolhoff, Lisa M.
Kobori, Y.
Koeniger, Gudrun
Koeniger, Nikolaus
Kohyama, Tetsuo I.
Krieger, Jürgen
Kryger, Per
Langridge, Keri
Lavine, Laura Corley
Lazo, Pedro A.
Lihoreau, Mathieu
Linn, Charles E.
Lorenz, Gus
Mafra-Neto, Agenor
Magori, Krisztian
Manoukis, Nicholas C.
Martel, Veronique
McAuslane, Heather
McNeil, Jeremy N.
Mercader, R. J.
Messing, Russell
Millar, Jocelyn
Miller, David
Milton, Katharine
Mller, Uli
Moore, Darrell
Mori, Nicola
Moribe, Hiroki
Müller, Caroline
Murchie, Archie
Nehring, Volker
Noge, Koji
Nuñez, Manuel Gonzalez
Oigiangbe, Nathaniel
Okutani, F.
Olson, Joelle f.
Ott, S. R.
Paine, Timothy
Paluzzi, Jean-Paul V.
Papadopoulos, Nikos
Park, Kye Chung
Peretti, Alfredo V.
Pozzebon, Alberto
Raine, Nigel
Raspotnig, Günther
Ratnadass, Alain
Reinecke, Andreas
Renkema, Justin M
Richmond, Douglas
Riley, David
Ritchie, Scott
Robinson, Willard S.
Roditakis, Emmanouil
Romero, Alvaro
Rust, Michael
Ruther, Joachim
Ryan, Geraldine D.
Sachse, Silke
Sánchez, Ismael
Sasaki, Tetsuhiko
Schafellner, Christa
Schäfer, Karina V. R.
Schetelig, Marc F.
Schneider, Stanley
Scott, David
Sezonlin, Michel
Shapiro, Arthur
Shvetsov, A. V.
Sokolowski, Michel
Springate, Simon
Srinivasan, Rajagopalbabu
Stansly, Phillip
Steidle, Johannes
Stelinski, Lukasz L.
Stökl, Johannes
Stout, Michael J.
Tan, Ken
Tena, Alejandro
Thany, Steeve
Thiéry, Denis
Tindall, Kelly V.
Tokuda, Gaku
Tsuji, Akihiko
Ueckermann, Eddie
Van Driesche, Roy
Vander Meer, Robert Kenneth
Vangansbeke, Dominiek
Vänninen, Irene
Vickers, Neil
Villanueva, Raul
Vogt, Heidrun
Walker, Robert J.
Wang, Changlu
Wang, Chen-Zhu
Wang, Minggang
Wanner, Kevin
Watanabe, Hirofumi
Webb, Cameron E
Williams, Ernest
Williamson, Chris
Witzgall, Peter
Wright, Denis
Yang, En-cheng
Yee, Wee L.
Zhu, Fang
Zhu, Feng
Zhu, Junwei J.
Zytynska, S. E.

